# Phylonium: fast estimation of evolutionary distances from large samples of similar genomes

**DOI:** 10.1093/bioinformatics/btz903

**Published:** 2019-12-02

**Authors:** Fabian Klötzl, Bernhard Haubold

**Affiliations:** Department of Evolutionary Genetics, Max-Planck-Institute for Evolutionary Biology, Plön, Germany

## Abstract

**Motivation:**

Tracking disease outbreaks by whole-genome sequencing leads to the collection of large samples of closely related sequences. Five years ago, we published a method to accurately compute all pairwise distances for such samples by indexing each sequence. Since indexing is slow, we now ask whether it is possible to achieve similar accuracy when indexing only a single sequence.

**Results:**

We have implemented this idea in the program phylonium and show that it is as accurate as its predecessor and roughly 100 times faster when applied to all 2678 *Escherichia coli* genomes contained in ENSEMBL. One of the best published programs for rapidly computing pairwise distances, mash, analyzes the same dataset four times faster but, with default settings, it is less accurate than phylonium.

**Availability and implementation:**

Phylonium runs under the UNIX command line; its C++ sources and documentation are available from github.com/evolbioinf/phylonium.

**Supplementary information:**

Supplementary data are available at *Bioinformatics* online.

## 1. Introduction

**Fig. 1.**
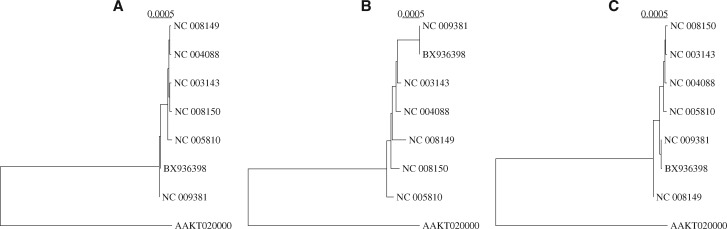
Alignment-based (**A**) and alignment-free (**B** and **C**) midpoint rooted neighbor-joining trees of eight *Yersinia* genomes. The alignment-free distances were computed using mash (Ondov *et al.*, 2016) (B) and phylonium (C)

Methods for rapid sequence comparison are a staple of bioinformatics, if not its raison d’être. Programs like FASTA and BLAST made the sequence data accumulated by molecular biologists navigable (Altschul *et al.*, 1997; Pearson, 1999). More recently, genome aligners like mugsy have allowed the comparison of whole genome samples (Angiuoli and Salzberg, 2011). For instance, the tree in Figure 1A of eight *Yersinia* genomes, each 5.3 Mb long, was computed by aligning them with mugsy in 7 min 23 s. The subsequent conversion of the alignment to a phylogeny was negligibly quick.

**Fig. 2.**
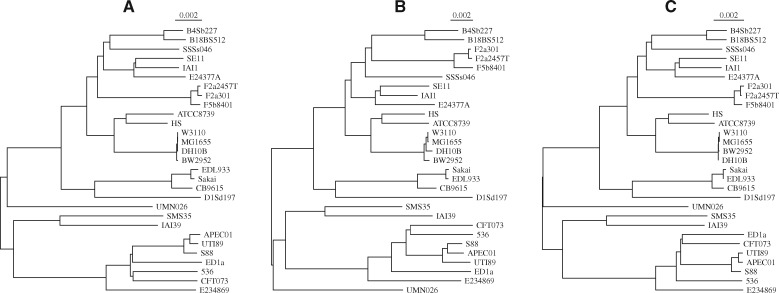
Alignment-based (**A**) and alignment-free (**B** and **C**) midpoint rooted neighbor-joining trees of 29 *E.coli*/*Shigella* genomes. The alignment-free distances were computed using mash (Ondov *et al.*, 2016) (B) and phylonium (C)

Similarly, the tree of 29 *Escherichia coli*/*Shigella* genomes with an average length of 4.9 Mb in Figure 2A is based on a mugsy alignment computed in 2 h 18 min. This large run time illustrates that genome aligners like mugsy do not scale well with sample size. However, distance matrices can be computed from genomes without first explicitly aligning all residues, leading to much faster methods of phylogeny reconstruction.

Zielezinski *et al.* (2019) recently reviewed 74 such ‘alignment-free’ methods implemented in 24 tools. Most of them rely on variants of exact matching to convert sequences directly to distances without prior alignment. These distances are then usually summarized into phylogenies with algorithms such as UPGMA or neighbor-joining (Felsenstein, 2004). Zielezinski *et al.* (2019) applied their collection of alignment-free distance methods to a battery of benchmarking datasets including the *Yersinia* and *E.coli*/*Shigella* samples shown in Figures 1 and 2. After ranking with respect to speed and accuracy, the winner was the program mash by Ondov *et al.* (2016).

Mash combines word-counting with clever mathematics to estimate substitution rates between genomes. For example, Figure 1B shows the neighbor-joining tree of the eight *Yersinia* strains based on mash distances. The tree is close to its alignment-based version and was computed in 2.5 s, that is 180 times faster than the alignment.

Röhling *et al*. (2019) observed that the distances returned by mash are affected by the addition of random regions. This is because mash distances are a function of the fraction of words shared between two sequences. This fraction is reduced by non-homologous regions leading to inflation of the distances; we show examples of this effect later.

Our program andi (Haubold *et al.*, 2015) for computing evolutionary distances between genomes is not affected by this problem because it is based on simplified local alignments. These are modeled by long maximal matches, the minimum length of which is computed from the distribution of match lengths in random sequences (Haubold *et al.*, 2009). Zielezinski *et al.* (2019) found that andi is among the faster of the tools surveyed—albeit much slower than mash—and highly accurate when applied to samples of closely related sequences. Such samples are becoming increasingly common as whole-genome sequencing is being used to monitor microbial epidemics, a development known as ‘genomic epidemiology’ (Tang *et al.*, 2017).

The aim of this study is to speed up andi while preserving its accuracy. In andi, substantial computational effort is spent on constructing an index in the form of an enhanced suffix array (Ohlebusch, 2013, chapter 4) for each sequence in the sample. These indexes are used to look up the exact matches on which the distance computation is based. This suggests the following speedup: index only a single reference sequence and pile all others onto it. This results in an approximate multiple sequence alignment, from which the desired distance matrix is calculated. We have implemented this idea in our new program phylonium.

Figure 1C shows the *Yersinia* tree based on phylonium distances. It was computed in 2.3 s, which is about as fast as mash (2.5 s). However, at a first glance the distances returned by phylonium are closer to the alignment tree than mash, especially near the tips. Similarly, Figure 2C shows the phylonium version of the *E.coli*/*Shigella* tree, which took 5.1 s to compute, 1600 times less than mugsy (8270 s). Again, it looks closer to the alignment tree than the mash tree computed in 9.4 s (Fig. 2B).

**Fig. 3.**
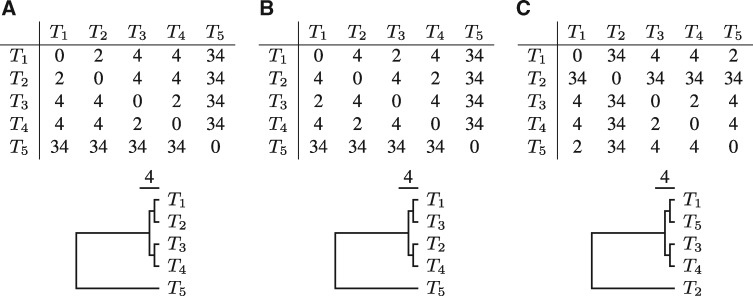
Three example distance matrices and the corresponding rooted trees. The Robinson–Foulds distances between trees **A**/**B** and **A**/**C** are 4. However, the distances between the matrices as defined by Equation (1) are Δ = 2 for **A**/**B** and Δ = 32 for **A**/**C**

In the following, the algorithm of phylonium is explained in more detail. Then, we compare the resource consumption and the accuracy of mash, andi and phylonium. When assessing accuracy, we follow the convention of using the Robinson–Foulds (RF) distance (Robinson and Foulds, 1981). However, alignment-free programs generate distance matrices, while the RF-distance quantifies topological differences between the trees computed from these distances. To clarify this distinction, consider the three distance matrices in Figure 3 and their corresponding trees. Between trees **A** and **B** taxa *T*_2_ and *T*_3_ have been swapped, leaving the clade $\left\{T_{1},\ldots ,T_{4}\right\}$ intact. In contrast, between trees **A** and **C** taxa *T*_2_ and *T*_5_ were swapped, which changes the quartet clade. Nevertheless, the RF-distance between **A** and **B** is the same as between **A** and **C**, 4. This contradicts our biological intuition that **A** is more similar to **B** than to **C**.

We therefore sought to also directly compare the distance matrices. An ideal measure should be simple and small for ‘similar’ matrices. The ‘Hausdorff’ distance between two sets, M,N is the largest distance between any pair of elements taken from M and N. This is used, for example, to measure distances between images—effectively matrices of pixels (Rucklidge, 1996). In pairs of sets, it is not known which pairs of elements correspond to each other. In contrast, the entries in distance matrices are labeled by pairs of taxon designations. So, we define as a Hausdorff-like distance between matrices the maximum difference between corresponding entries:

(1) $$\Delta =\max\{|D_{i,j}-d_{i,j}|:1\leq i,j\leq n\},$$

where *n* is the number of taxa. Now the distance between **A**/**B** in Figure 3, Δ = 2, is much less than Δ = 32 between **A**/**C**, reflecting our biological intuition about these three trees.

Still, distance matrices are hard to visualize and the point of their computation is usually phylogeny reconstruction. To combine the comparison of matrices and tree shapes, we simulate the datasets for assessing program accuracy along the *Yersinia* and *E.coli*/*Shigella* trees in Figures 1A and 2A using the program seq-gen (Rambaut and Grassly, 1997). As a final test, we apply the programs to all 2678 *E.coli* genomes in ENSEMBL. We find that phylonium preserves the accuracy of andi but is much faster. Compared to mash with default settings, it is slower in many situations but more accurate.

## 2. Materials and methods

### 2.1 Approximating the multiple sequence alignment

**Fig. 4.**
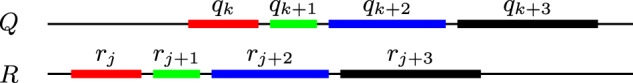
Anchors are long, equidistant, maximal matches between a reference, *R*, and some other sequence, *Q*. They form the basis of the computation of anchor distances, which in the end are converted to an estimate of the number of substitutions per site (Haubold *et al.*, 2015)

Phylonium constructs local alignments from exact matches that cannot be extended. Figure 4 shows four such unextendable, or *maximal*, matches between two sequences, the reference, *R*, and another element of the sample of *n* sequences, *Q*. Since the matches are maximal, they are flanked by mismatches. These mismatches are judged to be homologous, that is, they are polymorphisms, if the bracketing matches are longer than expected by chance as modeled by the null distribution of match lengths in random sequences (Haubold *et al.*, 2009). Say, the two leftmost matches in Figure 4, *r_j_* and *q_k_*, are longer than expected by chance; then they are called *anchors*. Further assume, the neighboring matches $r_{j+1}$ and $q_{k+1}$ are also anchors. If the physical distance between *r_j_* and $r_{j+1}$ is identical to that of matching pair $q_{k},q_{k+1}$, the anchors are concatenated into an approximate local alignment. In Figure 4, the four anchor pairs are equidistant and thus form a single local alignment. A pair of genomes would result in a large number of such approximate local alignments. Haubold *et al.* (2015) spell out the algorithm for finding these alignments in detail.

An extra complication is introduced by repeats, which can lead to multiple overlapping matches. These are resolved by picking the match that maximizes the number of aligned nucleotides using a chaining procedure described by Ohlebusch (2013, Section 8.3).

The number of mismatches bracketed by anchors, divided by their total length, estimates the number of mismatches per site. This is converted into the final number of substitutions per site using the Jukes–Cantor equation (Jukes and Cantor, 1969).

Phylonium piles the anchors of all *n* − 1 sequences onto *R*. This results in an approximate multiple sequence alignment used to compute all pairwise distances.

*Implementation* A central part of phylonium is the construction of the suffix array, the basis of the *enhanced* suffix array used in exact matching. Suffix array construction is delegated to the fast libdivsufsort library described by Fischer and Kurpicz (2017). The underlying divSufSort algorithm sorts alphabetically all suffixes of a string length ℓ in time O(ℓ log ℓ). The single enhanced suffix array built by phylonium from the reference sequence is then used to look up matches in all other input sequences in parallel.

The computation of Δ according to Equation (1) is implemented in the program mattools available from the same github page as phylonium.

### 2.2 Evaluating the multiple sequence alignment

The distances computed by phylonium vary with the underlying multiple sequence alignment, which in turn is sensitive to the reference chosen. To score a given multiple sequence alignment, we count the number of aligned nucleotides, which should be as large as possible. Our heuristic for achieving this is to use a ‘typical’ member of the sample as reference by choosing the genome of median length. Throughout this study, the reference sequence is always chosen according to this criterion. However, the user can set an arbitrary reference and observe the effect this has on the number of nucleotides aligned.

### 2.3 Data

Three datasets are analyzed in this study, eight *Yersinia* genomes, 29 *E.coli*/*Shigella* genomes, and all 2678 *E.coli* genomes in ENSEMBL, release 44. The *Yersinia* and *E.coli*/*Shigella* sets are part of the benchmarking data supplied by Zielezinski *et al.* (2019). Their URLs are listed in the Supplementary Information, which also contains instructions for downloading the *E.coli* genomes.

### 2.4 Measuring time and memory consumption

Resource consumption was measured on a computer equipped with 32 GB RAM and Intel Xeon CPUs for 24 cores running at 2.6 GHz under the Linux distribution Ubuntu 18.04. The three programs tested, mash version 2.1.1, andi version 0.13-beta and phylonium version 1.0, are all parallelized. However, unless stated otherwise, time measurements refer to the actual time elapsed in single-thread mode.

## 3. Results

### 3.1 Time and memory consumption

**Fig. 5.**
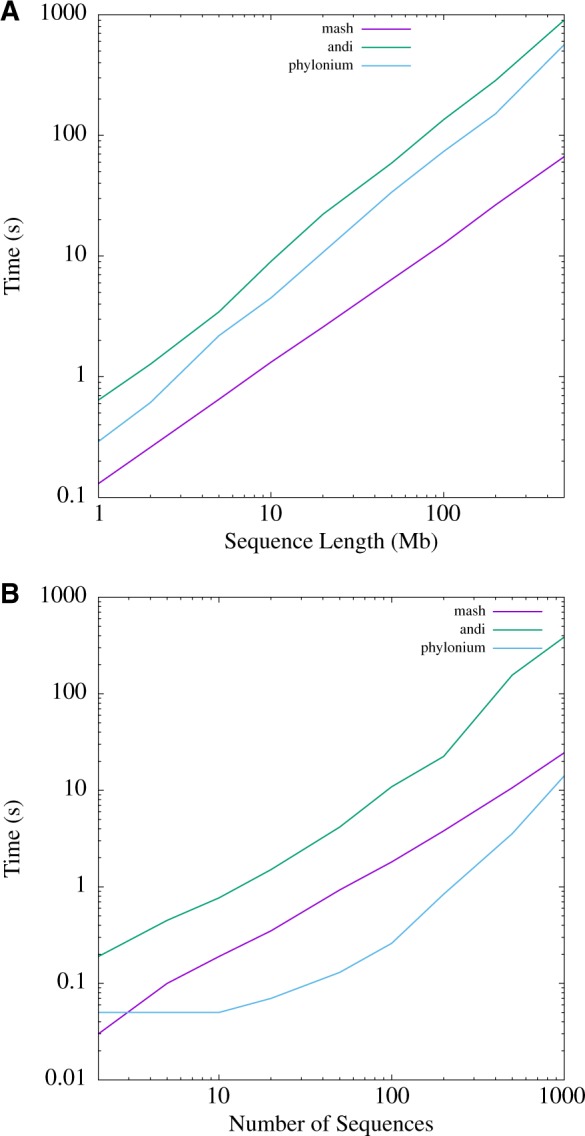
Time consumption of mash, andi and phylonium as a function of sequence length (**A**) and the number of 200 kb sequences (**B**)

Time and memory consumption were measured as a function of sequence length and sequence number. Sequence length was explored by simulating single pairs of sequences separated by 1% divergence. Mash analyzed a 500 Mb pair in 67 s, phylonium took eight times longer (565 s) and andi 13 times (902 s). Moreover, Figure 5A shows that the run time of mash grows more slowly than that of andi and phylonium as a function of sequence length.

When analyzing multiple 200 kb sequences, time consumption of mash is again less steep than that of andi (Fig. 5B). This is also true of phylonium, though it is actually faster than mash on these sets of relatively short sequences.

Memory consumption is almost linear in sequence length for the three programs tested (Supplementary Fig. S1A). However, mash used only 2.0 GB for a pair of 500 Mb sequences, while andi needed 14.7 GB and phylonium 22.0, that is eleven times more than mash.

As a function of the number of sequences, the memory requirement of mash is almost flat, while that of andi and phylonium behaves similarly with a steeper slope (Supplementary Fig. S1B).

### 3.2 Accuracy

Efficiency is only useful if combined with accuracy. In this section, the accuracy of phylonium is therefore explored with respect to the choice of reference sequence, the presence of random sequences, and diversity.

**Fig. 6.**
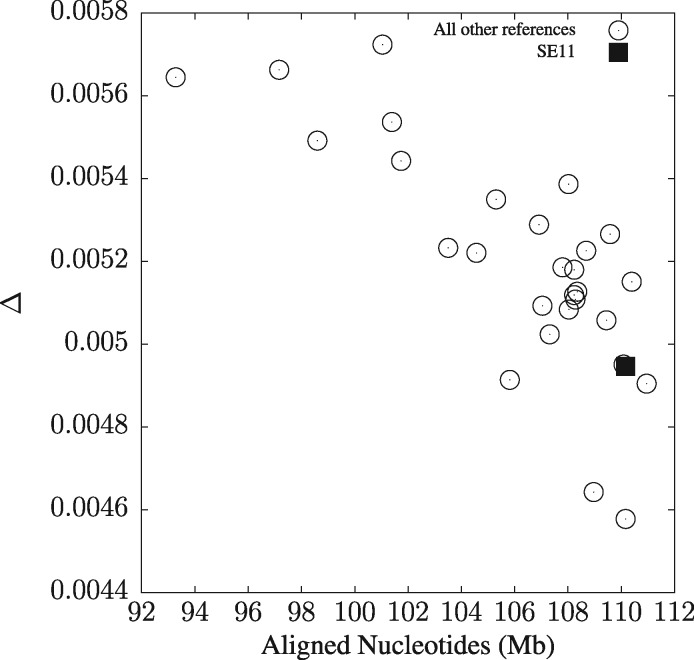
The difference between the true distances and those computed using phylonium, Δ, as a function of the number of aligned nucleotides for each of the 29 possible reference sequences

*Reference* Figure 6 shows the error measure, Δ, defined in Equation (1) as a function of the number of nucleotides aligned for each of the 29 possible reference sequences in the *E.coli*/*Shigella* dataset (Fig. 2). There is a significant correlation between Δ and the number of aligned nucleotides, r=−0.78, $P<10^{-6}$, so the aim should be to pick the reference that maximizes the number of aligned nucleotides. Our heuristic for doing this is to choose the genome with median length. This is strain SE11 with 4.9 Mb, which does indeed induce a high number of aligned nucleotides and the corresponding Δ is among the better ones.

*Unrelated regions* When comparing two 9 kb sequences, *S*_1_ and *S*_2_, separated by 0.01 substitutions per site, mash, andi and phylonium accurately estimate that distance (Supplementary Fig. S2). However, when *S*_2_ is augmented by 1 kb, or 10%, of random nucleotides, the mash distance grows from 0.01 to 0.012. At the same time, the mash*P*-value remains maximally significant, that is, zero. We repeated the addition of random 1 kb fragments until 50% of the sequence were random. The mash distance climbed continuously, eventually tripling to 0.03 (Supplementary Fig. S2). Phylonium and andi ignore non-homologous regions and the distances computed by them thus stayed at 0.01.

**Fig. 7.**
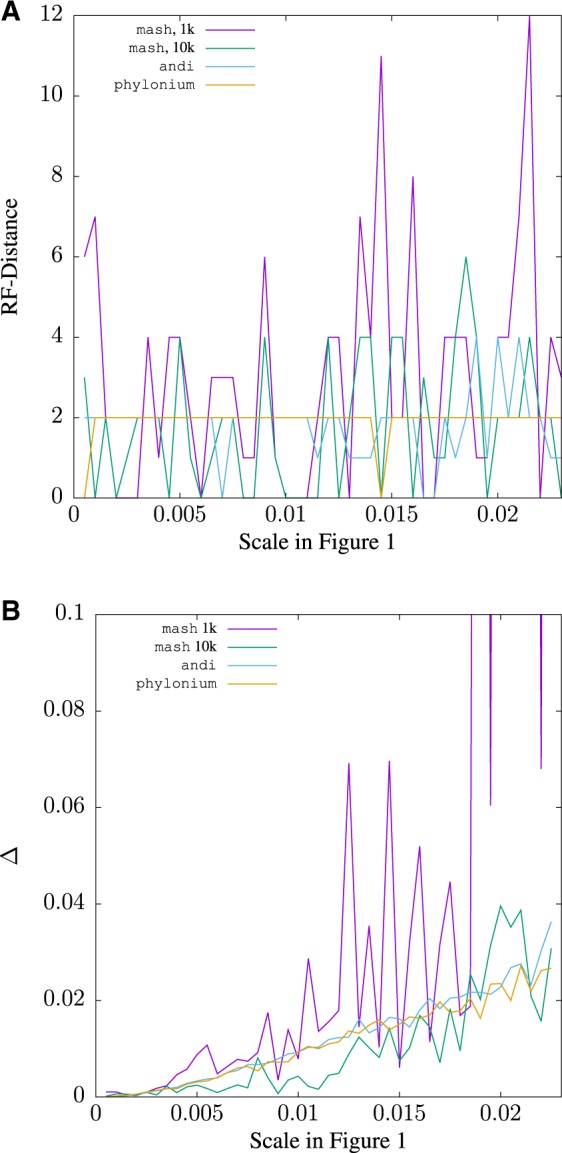
The Robinson–Foulds (RF) distance (**A**) and the difference, Δ (**B**), between the *Yersinia* tree in Figure 1A and a tree estimated from 200 kb sequences evolved along that tree as a function of the scale, which in Figure 1A is 0.0005 substitutions per site, the leftmost point on the graphs

*Diversity* A set of eight 200 kb sequences was simulated along the *Yersinia* tree in Figure 1A. The distances computed from these sequences were converted to a neighbor-joining tree and compared to the true tree. The original scale of 0.0005 corresponds to the leftmost point in Figure 7A and shows that the RF-distance of the mash tree is 6, that of andi 2, and of phylonium 0.

The *Yersinia* tree in Figure 1A can be ‘stretched’ by setting the scale bar to larger values, and the RF-distance recomputed. Figure 7A shows RF-distances as a function of the scale bar length. With default sketch size of 1000 (1k), the mash results fluctuate quite strongly. This is dampened with 10-fold larger sketches (10k). The topologies returned by phylonium and andi are closer to the true tree and fluctuate less. The simulations were carried out for a scale of up to 0.023, as for greater scales phylonium issued a warnings that distances were computed based on less than 20% aligned nucleotides. A scale of 0.023 roughly corresponds to a maximum distance of 0.35 substitutions per site.

Instead of comparing tree topologies, which may be misleading as demonstrated in Figure 3, distance matrices can also be compared directly using the maximum difference between corresponding entries, Δ, as defined in Equation (1). To first gain an intuition about the behavior of Δ, we simulated pairs of 200 kb sequences along the *Yersinia* tree in Figure 1A under three scenarios: simulate both datasets along the original tree, simulate one dataset along the original tree, the other along a tree where two taxa were switched, and simulate along two trees with the same branching pattern and lengths as the original tree but with shuffled taxon designations. As shown in Supplementary Figure S3A, these three simulation scenarios result in distinct Δ distributions. Similar results were obtained for the *E.coli*/*Shigella* tree (Supplementary Fig. S3B). This reassured us that Δ is useful for quantifying topological differences between phylogenies by directly comparing distance matrices.

When Δ was used to compare the tools investigated, andi and phylonium gave very similar results, which were better than those obtained with mash and default sketches for sequences evolved along the *Yersinia* tree (Fig. 7B). At a scale of 0.016, the mash curve jumps off the graph as the intersection between sketches is empty, which is encoded as a distance of 1. The accuracy of mash improves beyond that of phylonium for closely related distances if the sketch size is increased 10-fold to 10^4^. Larger sketches also extend the range of the program to greater distances.

**Fig. 8.**
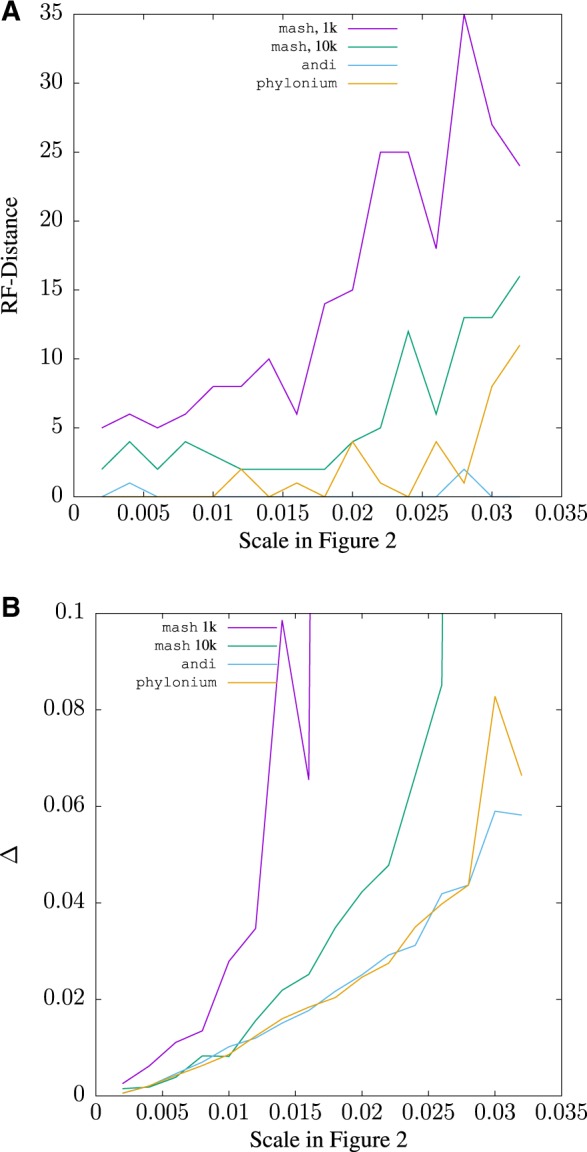
The Robinson–Foulds (RF) distance (**A**) and the difference, Δ (**B**), between the *E.coli*/*Shigella* tree in Figure 2A and a tree estimated from 200 kb sequences evolved along that tree as a function of the scale, which in Figure 2A is 0.002 substitutions per site, the leftmost point on the graphs

Similar observations were made when simulating sequences along the *E.coli*/*Shigella* tree in Figure 2A. Here, phylonium and andi gave better RF-distances across the full range of simulated divergence values (Fig. 8A). In addition, the distance matrices generated by andi and phylonium were equally close to the standard, except for the more divergent samples, where andi outperformed phylonium (Fig. 8B). The Δ-values for both programs remained below that of mash for 1k sketches. With 10k sketches the accuracy of mash was equal to that of phylonium for closely related sequences and then deteriorated until no distances were returned any more. This complete loss of homology signal happens earlier in mash than phylonium—the largest scale of 0.03 on the *E.coli*/*Shigella* tree corresponds to a maximum pairwise distance of 0.48 substitutions per site. However, further increases in sketch sizes are bound to improve the accuracy of mash further.

### 3.3 Application to real data

It took phylonium 1 h (3594 s) to analyze the 2678 *E.coli* genomes contained in the genomes collection of ENSEMBL. This is four times slower than the 982 s used by mash but 115 times faster than andi’s 115 h (412 786 s).

**Fig. 9.**
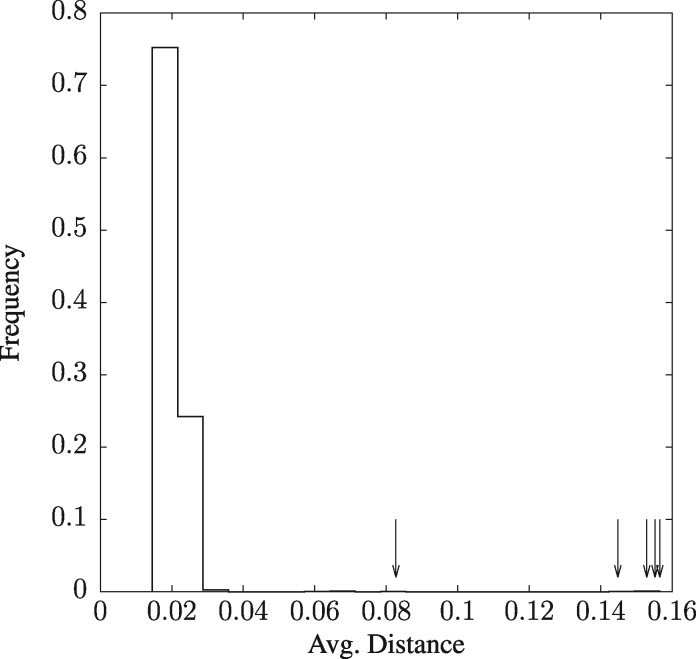
The distribution of the average number of substitutions per site (*distance*) computed by phylonium for the 2678 *E.coli* genomes contained in ENSEMBL. Arrows indicate the non-*E.coli* strains listed in Supplementary Table S1

It is difficult to inspect of a tree of 2678 taxa. Instead, we calculated the average distance of each strain to all other strains according to phylonium. As shown in Figure 9, the distance distribution contains a number of outliers beyond 0.04 substitutions per site. We suspected some of these might not be *E.coli* and investigated their identity by blasting the first couple of hundred bases in their sequence files. The five most extreme strains, highlighted by arrows in Figure 9, were indeed not *E.coli*. Supplementary Table S1 lists their original strain designation according to ENSEMBL and their ‘true’ taxonomy according to the BLAST website, which ranges from *E. albertii* to *Klebsiella pneumoniae*.

## 4. Discussion

The program presented here, phylonium, is a faster version of our published program andi (Haubold *et al.*, 2015). It is not the fastest tool for estimating reasonable genome distances, mash is still substantially faster for long pairs of sequences (Supplementary Fig. 5A). Moreover, in contrast to phylonium, mash, like a more recent version of its approach, skmer (Sarmashghi *et al.*, 2019), can be applied to unassembled reads (Ondov *et al.*, 2016). If we had taken assembly time into account, the speed advantage of mash would have been even greater. However, phylonium is particularly fast when applied to large samples (Supplementary Fig. 5B) and is more accurate than mash when applied to sequences where homology is only local (Supplementary Fig. S2). Andi and phylonium are not the only fast sequence comparison tool that reliably ignore nonhomologous regions; FastANI is a widely used alternative (Jain *et al.*, 2018), though it is slower than mash and slightly less accurate than phylonium (not shown). As the authors of mash point out, the accuracy of their tool improves with sketch size, and we show this by going from 1k sketches to 10k in Figures 7 and 8. Figure 7B also shows that mash misestimates distances as the intersection between sketches is reduced due to divergence. A similar problem occurs when phylonium is applied to divergent sequences: it cannot find any anchors and hence cannot estimate the distance. This restriction to closely related sequences makes phylonium suitable for applications like genomic epidemiology, but not as a general tool for estimating phylogenetic distances.

Nevertheless, we believe the push for speed by evolving andi into phylonium is worthwhile for two reasons: first, fast tools can become building blocks for other tools. For example, the multiple genome aligner mugsy (Angiuoli and Salzberg, 2011) used in this study is built on the pioneering MUMmer package for pairwise genome alignment (Delcher *et al.*, 1999; Kurtz *et al.*, 2004). Phylonium might be used, for instance, to rapidly compute guide trees in conventional multiple sequence aligners. Secondly, there is a well-known trade-off between computing and storage. A multiple sequence alignment of 2678 *E.coli* genomes would contain 13.9 Gb plus gaps. Phylonium approximates this unwieldy structure so rapidly, recomputation becomes more convenient than storage.

The speed of phylonium is achieved by the old idea to pile all sequences in a sample onto a single reference. This works reasonably well because genomes contain so much information that the loss of homologous regions due to the quirks of a particular reference are often negligible resulting in a small range of Δ values when varying the reference in the *E.coli*/*Shigella* sample (Fig. 6). In addition, speed is achieved by parallelization. We have not explored this aspect here to concentrate on the algorithms, but in practice parallelization is important. On the 24 core test machine, phylonium in parallel mode took 12 min 24 s to analyze the 2678 *E.coli* genomes, compared to 3 min 21 s used by mash. This is still a 4-fold speed difference, as was already observed in single-thread mode. Mash and phylonium identified the same outliers, and the fact that five of these genomes turned out to not even be *E.coli* (Supplementary Table S1), demonstrates that speed can aid discovery.

Speed should not reduce accuracy too much, though. When measuring accuracy, we propose to compare distance matrices directly using Equation (1) (Figs 7B and 8B). Qualitatively this gives similar results as obtained by the traditional RF-distance (Figs 7A and 8A) and both metrics showed the greater accuracy of phylonium compared to mash with default sketches. With 10-fold larger sketches, mash was more accurate than phylonium on low divergence data (Figs 7B and 8B), while phylonium was more accurate when divergence was increased (Fig. 8B). However, there are situations where the RF-distance hides important discrepancies between trees (Fig. 3). Our alternative, Δ, is easy to read from two matrices but still effectively reflects the difference between two trees, because it is small exactly if they are similar (Supplementary Fig. S3).

Since phylonium is based on the same anchor distances as andi, the accuracy of the two programs is similarly high (Figs 7 and 8) while phylonium is much faster when applied to large datasets (Fig. 5B). Given that genomics looks set to become the norm in epidemiology (Tang *et al.*, 2017), phylonium may serve as an accurate and efficient alternative to mash in this field.

## Supplementary Material

btz903_Supplementary_Information
