# Supplementary material for: Phylonium: fast estimation of evolutionary distances from large samples of similar genomes
Source: Bioinformatics. 2019 Dec 2;36(7):2040–6. doi: 10.1093/bioinformatics/btz903 (PMC7141870; doi:10.1093/bioinformatics/btz903)
Supplement: btz903_Supplementary_Information [file btz903_supplementary_information.pdf]

Supplementary Information for `Phylonium`: *Fast Estimation of Evolutionary Distances from Large Samples of Similar Genomes*

Fabian Klötzl & Bernhard Haubold
Department of Evolutionary Genetics, Max-Planck-Institute for Evolutionary Biology, Plön, Germany

October 30, 2019

Data Sets

Three data sets are analyzed in this paper, eight *Yersinia* genomes, 29 *E. coli*/*Shigella* genomes, and all *E. coli* genomes in ENSEMBL. These are downloaded as follows:

- Eight *Yersinia* genomes

  ```
  wget afproject.org/media/genome/hgt/unsimulated/yersinia/dataset/x
  ```

  where `x` is

  ```
  unsimulated-yersinia.zip
  ```

- 29 *E. coli*/*Shigella* genomes

  ```
  wget afproject.org/media/genome/std/assembled/ecoli/dataset/y
  ```

  where `y` is

  ```
  assembled-ecoli.zip
  ```

- All *E. coli* genomes in ENSEMBL
  - Download the names of all species in the ENSEMBL genome collection

    ```
    wget ftp://ftp.ensemblgenomes.org/pub/current/species.txt
    ```

  - Extract the genomes of *E. coli* and generate the corresponding commands, which are saved to a download script

    ```
    grep 'Escherichia coli' species.txt |
    awk -F '\t' -f printWget.awk > getEco.sh
    ```

    where `printWget.awk` is

    ```
    {
        collection = $13
        gsub("collection_.*", "collection", collection)
        name1 = $2
        name2 = $2
        sub("^e", "E", name2)
        cmd = "wget ftp://ftp.ensemblgenomes.org/pub/current/bacteria/fasta/" \
              collection "/" name1 "/dna/" name2 "*.dna.toplevel.fa.gz"
        print cmd
    }
    ```

  - Download sequences

    ```
    bash getEco.sh
    ```

Memory Consumption

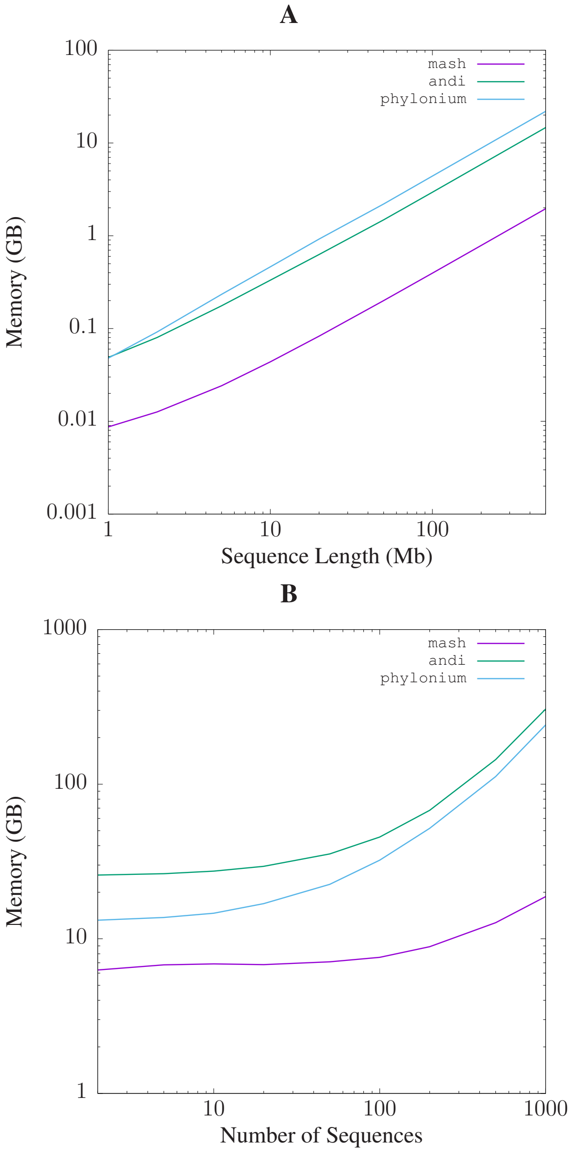

Figure S1: Memory consumption of `mash`, `andi`, and `phylonium` as a function of sequence length (**A**) and number of 200 kb sequences (**B**).

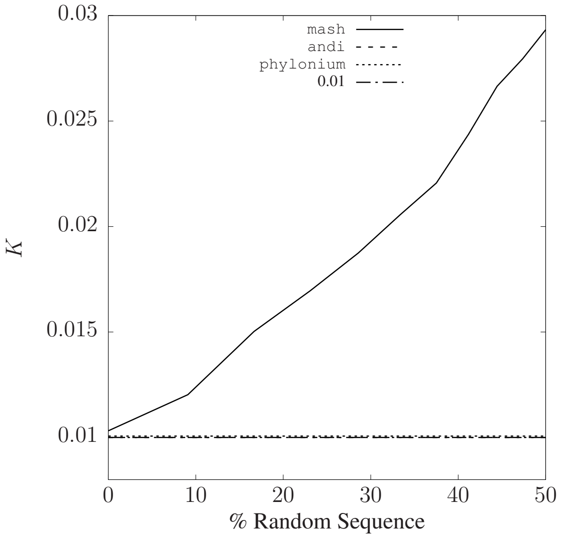

Figure S2: The effect of random regions on distance estimation. Initially, S_1 and S_2 consist of 9 kb separated by $K = 0.01$ substitutions per site. S_2 is then augmented by random 1 kb regions. The true distance, $K = 0.01$ and the results of `andi` and `phylonium` are indistinguishable.

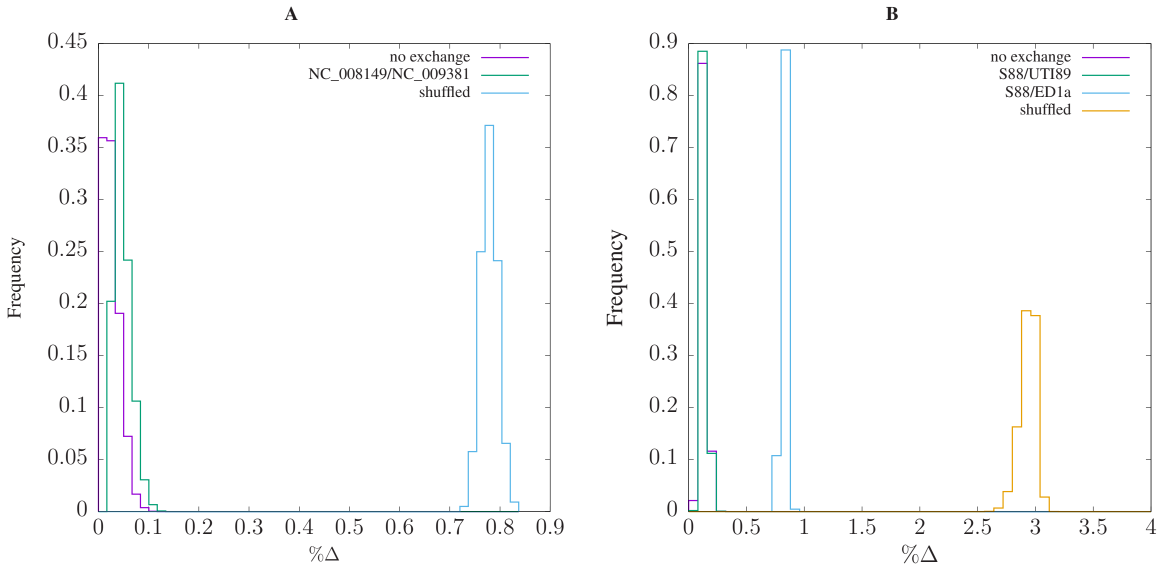

Figure S3: Distribution of Δ values when simulating 10^4 pairs of 200 kb sequences along the *Yersinia* (**A**) or the *E. coli*/*Shigella* (**B**) tree in Figures 2A and 3A, respectively. The annotations indicate which strains were exchanged; *shuffled* means taxa names were distributed randomly among the tips of the tree.

Table S1: Apparently mislabeled *E. coli* strains contained in ENSEMBL

#	Original	True
1	GCA_001286085	*Escherichia albertii*
2	ISC11	*Citrobacter freundii*
3	ISC56	*Klebsiella pneumoniae*
4	GCA_001443095	*Enterobacter hormaechei*
5	GCA_900092915	*Klebsiella pneumoniae*
